# Study protocol ROTATE-trial: anterior cruciate ligament rupture, the influence of a treatment algorithm and shared decision making on clinical outcome– a cluster randomized controlled trial

**DOI:** 10.1186/s12891-021-04867-5

**Published:** 2022-02-05

**Authors:** Floris H. de Vos, Duncan E. Meuffels, Marleen de Mul, Marjan Askari, Erwin Ista, Suzanne Polinder, Erwin Waarsing, Sita M. Bierma-Zeinstra, Max Reijman, on behalf of the ROTATE study group, E. R. A. van Arkel, R. W. Brouwer, A. R. J. Langeveld, R. Riedijk, J. A. C. Zijl, R. P. A. Janssen, D. J. Hofstee, R. G. Zuurmond, M. A. van Rhee

**Affiliations:** 1 Department of Orthopaedics and Sports Medicine, Erasmus MC University Medical Center, Rotterdam, The Netherlands; 2 Erasmus School of Health Policy & Management, Erasmus University Rotterdam, Rotterdam, The Netherlands; 3 Department of Internal Medicine, section Nursing Science, Erasmus MC University Medical Center, Rotterdam, The Netherlands; 4 Department of Public Health, Erasmus MC University Medical Center, Rotterdam, The Netherlands; 5 Department of General Practice, Erasmus MC University Medical Center, Rotterdam, The Netherlands

**Keywords:** Anterior cruciate ligament rupture, Treatment algorithm, Shared decision making, Cost-effectiveness

## Abstract

**Background:**

Anterior cruciate ligament (ACL) rupture is a very common knee injury in the sport active population. There is much debate on which treatment (operative or non-operative) is best for the individual patient. In order to give a more personalized recommendation we aim to evaluate the effectiveness and cost-effectiveness of a treatment algorithm for patients with a complete primary ACL rupture.

**Methods:**

The ROTATE-trial is a multicenter, open-labeled cluster randomized controlled trial with superiority design. Randomization will take place on hospital level (*n* = 10). Patients must meet all the following criteria: aged 18 year or older, with a complete primary ACL rupture (confirmed by MRI and physical examination) and maximum of 6 weeks of non-operative treatment. Exclusion criteria consists of multi ligament trauma indicated for surgical intervention, presence of another disorder that affects the activity level of the lower limb, pregnancy, and insufficient command of the Dutch language. The intervention to be investigated will be an adjusted treatment decision strategy, including an advice from our treatment algorithm. Patient reported outcomes will be conducted at baseline, 3, 6, 12 and 24 months. Physical examination of the knee at baseline, 12 and 24 months. Primary outcome will be function of the knee measured by the International Knee Documentation Committee (IKDC) questionnaire. Secondary outcomes are, among others, the Tegner activity score, the Knee injury and Osteoarthritis Outcome Score (KOOS) and the 9-item Shared Decision Making Questionnaire (SDM-Q-9). Healthcare use, productivity and satisfaction with ((non-)operative) care are also measured by means of questionnaires. In total 230 patients will be included, resulting in 23 patients per hospital.

**Discussion:**

The ROTATE study aims to evaluate the effectiveness and cost-effectiveness of a treatment algorithm for patients with a complete primary ACL rupture compared to current used treatment strategy. Using a treatment algorithm might give the much-wanted personalized treatment recommendation.

**Trial registration:**

This study is approved by the Medical Research Ethics Committee of Erasmus Medical Center in Rotterdam and prospectively registered at the Dutch Trial Registry on May 13th, 2020. Registration number: NL8637.

## Background

Anterior cruciate ligament (ACL) rupture is a very common knee injury in the sport active population and has been well researched over the years. There is much debate on which treatment is best for the individual patient. We describe two treatments for an ACL rupture, namely anterior cruciate ligament reconstruction (ACLR) (operative treatment) and an intensive rehabilitation program (non-operative treatment). The primary goal of both treatments is to prevent instability of the knee joint. If treated non-operatively, the patient is guided through a rehabilitation program. If treated operatively, the patient receives a reconstructed ACL.

Two large multi-center randomized controlled trials, the COMPARE-study and the KANON-study, conclude that early (within 6 weeks of trauma) operative treatment of an ACL rupture is not superior to non-operative treatment plus optional delayed (after 3 months of trauma) ACLR [1, 2]. Half of the patients from both trials treated initially non-operatively were reconstructed during the 2 years follow-up. This means that this group eventually had two rehabilitation periods, namely one prior and one after the ACLR. It also suggests that half of the patients treated initially operatively, might not need surgical intervention. In the cost-effectiveness analysis of the COMPARE study, we reported that patients treated with rehabilitation and optional delayed ACLR have the lowest gain in quality of life and highest costs compared to patients treated with rehabilitation alone [3]. Segregation of patients at an early stage that perform well with non-operative treatment from those who do not, might influence the gain in quality of life and decrease costs on public healthcare by decreasing the proportion of patients who have two rehabilitation periods. Current practice is that treatment recommendation is based on expert opinion, taking into account patient characteristics and preferences (e.g. operative treatment because of an active sporting career) [4]. To give an appropriate and patient tailored answer to the question what to do with a patient with an ACL injury on individual level, we constructed a treatment algorithm to be used in the shared decision making process between physician and patient.

Treatment algorithms are nowadays widely used throughout medical healthcare [5]. The advantage of algorithms is the ability to analyze diverse data types (demographic data, laboratory findings, imaging data, and doctors’ free-text notes) and incorporate them into predictions for disease risk, diagnosis, prognosis, and appropriate treatments on an individual level [5]. Using a treatment algorithm for patients with ACL injuries might give the much-wanted personalized treatment recommendation and enables identification of the group of patients who perform poorly despite (non-)operative treatment.

In the decision-making process, scientific evidence, clinical expert opinion, and patient values or preferences come together to reach a shared decision about rehabilitation or surgery. There are some tools, such as decision aids or consultation cards that aim to facilitate this process [6]. Using a treatment algorithm might improve the perceived shared decision making, because the recommendations become more personalized. The effect of treatment algorithms and shared decision making on functional outcome of the knee after ACL rupture, to our knowledge, has not yet been researched.

This research topic was prioritized within the research agenda of the Dutch Orthopaedic Association (NOV).

## Methods

This manuscript is written according to the Consolidated Standards for Reporting Trials (CONSORT statement) and Standard Protocol Items: Recommendations for Interventional Trials (SPIRIT guidelines [7, 8].

### Objectives

The aim of the current project is to evaluate the effectiveness and cost-effectiveness of the treatment algorithm for patients with a complete primary ACL rupture compared to current used treatment strategy. We hypothesize that the treatment algorithm will lead to earlier identification of the optimal treatment for the individual patient. We expect that fewer patients will receive a surgical reconstruction compared to the current practice and that fewer patients will need delayed surgery after non-operative treatment has failed. Hence, we hypothesize that with the treatment algorithm faster recovery of functional outcome will be achieved compared to the current practice (superiority study). As secondary outcome we expect that a faster recovery of functional outcome will lead to a faster return to work and sports. Hence, we hypothesize that the treatment algorithm will lead to lower societal cost. The treatment algorithm provides extra information for the physician and as well for the patient, to include in their decision for a(n) (non-)operative treatment. Therefore we expect the treatment algorithm will impact the shared decision making communication process.

#### Primary objective

- Assessing whether the treatment algorithm for patients with a complete primary ACL rupture is superior to the current practice regarding recovery of functional outcome at two-year follow-up.

#### Secondary objectives

- Assessing whether the treatment algorithm for patients with a complete primary ACL rupture is superior to the current practice regarding recovery of functional outcome during two-year follow-up.
- Assessing whether this treatment algorithm will increase the cost-effectiveness compared to the current practice.
- Assessing whether this treatment algorithm will lead to less ACL reconstructions compared to the current practice.
- Assessing whether this treatment algorithm will extend the (perception of) shared decision making during a physician-patient consultation.

### Study design

The ROTATE-trial is an open-labeled cluster randomized controlled trial with superiority design. It is a multicenter study performed in orthopedic departments of ten hospitals throughout the Netherlands. Of the ten hospitals one is an academic hospital, one a private clinic and eight are general hospitals. In all participating hospitals, the local responsible authorities approved the conduct of the study. The study protocol was approved by the Erasmus MC Ethics Committee and was registered in the Dutch Trial Registry. (Registration number: NL8637).

### Recruitment procedures

All adults with an ACL rupture, visiting an orthopedic surgeon of one of the participating hospitals, who meet the eligibility criteria will be invited to participate. These patients receive the participant information sheet and will have sufficient amount of time to decide upon participation. Written informed consent is obtained from the patient prior to inclusion. At inclusion, the patient and physician will come to a treatment preference dependent on which treatment decision strategy they will use.

### Inclusion criteria

In order to be eligible to participate in this study, a subject must meet all the following criteria: aged 18 year or older, with a complete primary ACL rupture (confirmed by magnetic resonance imaging (MRI) and physical examination), maximum of 6 weeks of non-operative treatment, and willing to comply with the study protocol.

### Exclusion criteria

A potential subject who meets any of the following criteria will be excluded from participation in this study: multi ligament trauma indicated for surgical intervention (eg. every multi ligament trauma besides medial collateral injury combined with an ACL rupture), presence of another disorder that affects the activity level of the lower limb (such as lateral/posterolateral ligament complex with significantly increased laxity, general systemic disease affecting physical function or any other condition or treatment interfering with the completion of the trial, including patients with metal devices or motion disorders), pregnancy, and insufficient command of the Dutch language.

### Randomization

To avoid dilution between both treatment decision strategies, randomization will take place on hospital level. This means that the treatment choice of all patients referred by one hospital will be based on the same treatment decision strategy. This will be done to overcome that the treatment choice of a participating orthopedic surgeon is influenced by knowledge of the algorithm, in case the patient is assigned to the control group. Secondly, this will be done to overcome logistic difficulties such as switching back and forth between different treatment decision strategies. Randomization will take place when institutional review board acceptance has been obtained, and will be executed by the use of a computer generated randomization list.

### Algorithm development

A treatment algorithm has been formulated based on current literature, results of the COMPARE study, and finalized by an expert group consisting of orthopaedic surgeons and researchers specialized in traumatic knee injuries [2]. Disagreements on the contents of the algorithm were resolved by consensus. This algorithm generates an advice which patients have a high probability that they a) will not respond to an exercise program under supervision of a physical therapist, and are therefore indicated for early surgical reconstruction and b) will respond to an exercise program. The algorithm application will be used in the shared decision process to come to a personalized treatment choice.

### Intervention group

**Fig. 1:**
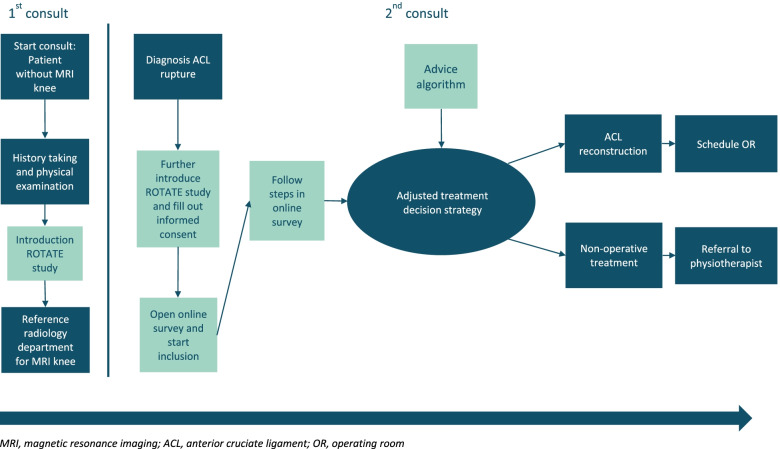
Flowchart adjusted treatment decision strategy intervention group. MRI, magnetic resonance imaging; ACL, anterior cruciate ligament; OR, operating room

At the initial consult, the ACL rupture will be clinically diagnosed. Additional patient specific information will be collected essential for the ACL treatment algorithm. The patient will be referred to the radiology department for an MRI of the affected knee. During the second consultation the physician and patient come to a shared treatment plan for treating ACL rupture based on multiple determinants, including an advice from the treatment algorithm. To ensure study methodological safety and because the ROTATE-trial is still including patients we can’t disclose what other determinants are added in the adjusted treatment decision strategy. The adjusted treatment decision strategy is visualized in Fig. 1.

### Control group

Usual care in which the treatment decision is made based upon the experience of the orthopedic surgeon combined with the preference of the patient.

### Measurements

Patients will be asked to fill in questionnaires at baseline, 3, 6, 12, and 24 months. Knee specific physical examination, based on the International Knee Documentation Committee (IKDC) examination form, will be performed at baseline, 12 and 24 months. Physical examination consists of the following: passive range of motion, presence of effusion, valgus / varus stress test, Lachman test, anterior drawer and pivot shift test. In each participating hospital the knee specific physical examination will be performed by an orthopedic surgeon or an assessor trained by the coordinating researcher.

### Shared decision making process

In this study, specific attention is given to shared decision making. This takes into account that not only the outcome of the decision is important (determination), but also the process of coming to a decision (deliberation) [9, 10].

The shared decision making process will be evaluated from the perspective of the patient and the physician. Patients will be asked for their treatment preference three times, as explained above in the description from the intervention group. Through this we can measure the patients preference before and after the intervention. After the second consult, the decisional process on patient level is measured by a questionnaire. In addition, the treatment preference of the physician is recorded for this study, without disclosing this information to the patient. The physician is allowed to share his or her treatment preference after the advice from the algorithm is given. The decisional process on physician level is measured qualitatively through focus groups in each participating center. For these sessions, which will be chaired by the researcher of the coordinating center, the surgeon specialized in treating ACL ruptures will be invited. In case only one physician participates in the study, an individual interview will be conducted instead. The focus groups will be conducted twice in the intervention group centers and once in the control group centers, unless there has been a change in treatment protocol in one of the control group centers. Focus groups/interviews will be audio recorded with permission. Patients’ and physicians’ remarks or scores on the shared decision making process will be evaluated and compared between intervention and control group centers.

## Outcomes

### Primary outcome

The primary outcome is the difference between both groups in patients’ perception of symptoms, knee function and ability to participate in sports activities assessed by IKDC questionnaire at 24 months follow-up. A higher IKDC score reflects more favourable patients’ ratings of symptoms, knee function, and ability to participate in sports activities (optimal score is 100). The IKDC is a valid and responsive (ability to detect changes in time) outcome measure for patients with an ACL rupture [11].

### Secondary outcomes

At baseline we will collect the following baseline characteristics, age, sex, height, weight, educational level, smoking habit, side affected and date and kinetics of trauma. Patient-rated outcome measures (PROMs) will be sent online at baseline, 3, 6, 12 and 24 months. There is an overview of all PROMs per follow-up moment in Table 1.

**Table 1:** Patient-rated outcome measures (PROM) per follow-up moment

*PROMs*	*Baseline/1st consult*	*3 months*	*6 months*	*12 months*	*24 months*
*IKDC - subjective*	*x*	*x*	*x*	*x*	*x*
*Tegner*	*x*	*x*	*x*	*x*	*x*
*KOOS*	*x*				*x*
*Lysholm*	*x*	*x*	*x*	*x*	*x*
*NRS*	*x*	*x*	*x*	*x*	*x*
*Tampa scale*	*x*			*x*	*x*
*EQ-5D-5L*	*x*	*x*	*x*	*x*	*x*
*SDM-Q-9*	*x*				
*iMCQ/iPCQ*		*x*	*x*	*x*	*x*

*PROM* patient-rated outcome measures, *IKDC* International Knee Documentation Committee, *KOOS* Knee injury and Osteoarthritis Outcome Score, *NRS* number rating scale, *EQ-5D-5L* EuroQol 5D, *SDM-Q-9* 9-ltem Shared Decision Making Questionnaire, *iMCQ* Medical

We will measure the patients’ perception of symptoms, knee function and ability to participate in sports activities at 3, 6 and 12 months follow-up with the IKDC questionnaire. As mentioned above, the 24 month follow-up will be our primary outcome. Work and sports activities will be measured with the Tegner questionnaire (range 0–10; highest activity score is 10) at baseline, 3, 6, 12 and 24 months [12]. The Knee injury and Osteoarthritis Outcome Score (KOOS) will be assessed at baseline and 24 months of the subscales pain, symptoms, ADL, sports and quality of life (QoL) (range 0–100; optimal score is 100) [13]. Knee instability will be measured with the Lysholm questionnaire (range 0–100; optimal score is 100) at baseline, 3, 6, 12 and 24 months [12]. Knee pain will be assessed with the number rating scale (NRS) (range 0–10; optimal score is 0) at baseline, 3, 6, 12 and 24 months. Kinesiophobia will be measured with the Tampa scale at baseline (range 17–68; optimal score is 17), 12 and 24 months [14]. General quality of life will be measured with the EQ-5D-5L questionnaire (range − 0.329 to 1; optimal score is 1) at baseline, 3, 6, 12 and 24 months. EQ-5D-5L use is recommended for the assessment of quality of life in trauma patients, especially for economic assessments. The patients’ EQ-5D-5L health states will be converted into utility scores using the Dutch tariff. The EQ-5D will be used for the cost-utility analysis [15].

Intramural and extramural medical costs will be assessed with the Medical Consumption Questionnaire (iMCQ) and productivity loss will be assessed with the Production Consumption Questionnaire (iPCQ) at 3, 6, 12, and 24 months follow-up. These questionnaires are validated by the Institute of Medical Technology Assessment (Erasmus University, Rotterdam, The Netherlands). iMCQ includes details on medical specialist care, physical therapy, hospitalization, nursing home, home care, and other costs directly associated with diagnosis, treatment and rehabilitation. iPCQ includes details on work resumption and production losses [16].

For patients, the efficacy of the shared decision making process will be evaluated using the score on the 9-ltem Shared Decision Making Questionnaire (SDM-Q-9) that is administered directly after the consultation. The SDM-Q-9 is a patient-reported measure that focuses on the decisional process by rating physicians’ and patients’ behavior in medical encounters [17]. The aggregated scores over all items of the SMD-Q-9 lead to a total raw score between 0 and 45, with 0 indicating the lowest and 45 indicating the highest level of perceived shared decision making.

For physicians, the efficacy of the shared decision making process will be evaluated at one or two time points, at the start of the inclusion period prior to the first inclusion in intervention and control centers and at the end of the inclusion period after the last inclusion in intervention centers. This will be evaluated using interviews/focus groups. The proposed topic lists are based on the SDM-Q-9-Doc questionnaire (the physician version of the SDM-Q-9) and the Technology Acceptance Model (TAM) [18]. Every interview/focus group session will be analyzed qualitatively and compared between control and intervention centers. After the inclusion period, we will analyze whether there is an improvement of perceived shared decision making when using our treatment algorithm.

Physical examination of the knee will be assessed by the objective IKDC questionnaire at baseline, 12 and 24 months. (Table 2) Information about the received treatment is reported including the surgeons fidelity to the intervention. We address the number of patients that cross over from their allocated treatment. (Serious) Adverse events ((S)AE’s) are noted.

**Table 2:** Measurements per follow up moment

	*Baseline*	*3 months*	*6 months*	*12 months*	*24 months*
*Physical examination*	*x*			*x*	*x*

### Sample size

Our primary research hypothesis is that with the treatment algorithm a better patients’ perception of symptoms, knee function and ability to participate in sports activities will be achieved compared to the current practice after 24 months (superiority study). Patients’ perception of symptoms, knee function and ability to participate in sports activities will be evaluated at multiple time points during 2 year follow-up, and will be expressed as change in IKDC score (subjective form) compared to baseline. The standard deviation (SD) of the IKDC after 24 months of follow-up has been reported to be 10,7. We aim to find a clinically relevant additional effect of the algorithm to usual care. Therefore, our hypothesis is that the potential additional effect size should be minimally 0.5 (6 points). For the intra cluster correlation (ICC) coefficient we used an ICC of 0.10 which is reported for hospital processes. Based on a difference of 6 points, SD of 10.7, an ICC of 0.10 (randomization on hospital level), a power of 90%, an α of 0.05, and 10 participating hospitals; 20 patients per hospital need to be included (200 patients in total). To accommodate a 10% dropout rate, 23 patients per hospital are required, resulting in a final sample of 230 patients (10 hospitals * 23 patients per hospital).

### Data analysis

#### Primary outcome

In our primary analysis patients will be analysed according to their randomization group. To answer our primary research question, we will use linear mixed models to evaluate the between group difference in patients’ outcome as assessed by the IKDC questionnaire. The IKDC score after 24 months follow-up will be used as dependent variable. The randomized allocation will be used as an independent variable. We will adjust the analysis for potential confounders, namely baseline IKDC score. The used cluster (hospital) will be added as random factor into the model. The following model assumptions will be checked: linearity, homoscedasticity and normality of residuals.

#### Secondary outcome

To evaluate the between group difference in course in IKDC scores over the follow-up period, as indicated by the interaction between time point and randomized allocation, we will use mixed models. The IKDC score (at baseline and after 3, 6, 12 and 24 months of follow-up) will be used as a dependent variable. The randomized allocation will be used as an independent variable. Follow-up period and the interaction between follow-up and randomized allocation will be entered into the model as fixed factors.

Difference between groups in knee pain, kinesiophobia, shared decision making for patients and quality of life will be used as secondary outcomes. The analyses will be performed by using linear or binary mixed models for repeated measures. Return to pre-injury sport level, satisfaction with treatment and adverse events will be reported as comparative frequencies. Because of the potential for type 1 error due to multiple comparisons, findings for analyses of secondary endpoints should be interpreted as exploratory.

Difference between groups in shared decision making for physicians will be analyzed qualitatively, using the transcripts of the interviews’/focus groups’ audio files. The coding process will result in themes and patterns of shared meaning, underpinned or united by a central concept which are important for our research question [19].

### Cost effectiveness analysis

An economic evaluation will be conducted from a societal perspective in accordance with the Dutch guidelines [20] in which medical costs and loss of productivity costs will be considered. The time horizon will be 2 years to include all relevant costs and effects.

Both cost-utility (CUA) and cost-effective (CEA) analysis will be executed. Direct intramural and extramural care costs will be calculated (e.g. operation, physiotherapy, hospital days, costs of side effects, wound infections) and indirect non-medical costs (e.g. productivity losses). Data on medical resource use will be collected from the electronic hospital information systems, based on the iMTA Medical Consumption Questionnaire (iMCQ). Productivity costs are assumed to be substantial and will be registered in detail by the iPCQ. See Table 1 for follow-up moments regarding these questionnaires.

Healthcare costs will be analyzed conform to charges published in Dutch guidelines as they are most representative for the real healthcare costs [20]. The unit price of the ACL reconstruction and the intensive exercise program will be calculated, per individual center, with the micro-costing method.

The economic evaluation of the ACL treatment algorithm for patients with an ACL rupture compared to usual care will be calculated as the incremental cost-effectiveness ratio (ICER). The change in recovery of physical functioning, as assessed by the IKDC, will be the primary effect outcome measure for the CEA and quality adjusted life years (QALY) for the CUA. QALYs will be calculated for a 2 year period, according to the Dutch tariff for the EQ-5D.

In order to account for the possible clustering of data, analyses will be performed using linear multilevel analyses [21]. Accounting for the possible clustering of data (e.g., at the hospital level) is of great importance, as most economic evaluations fail to do so, whereas ignoring the possible clustering of data might lead to inaccurate levels of uncertainty and inaccurate point estimates.

The stability of the results to changes in costs and effect parameters will be measured with a sensitivity analysis. We will use bootstrapping with 5000 replications to determine 95% confidence intervals around the uncertainty surrounding ICERs and cost differences. Cost effectiveness planes and acceptability curves will be graphically presented using the net benefit framework [22]. Cost-effectiveness acceptability curves show the probability that the intervention is cost-effective in comparison with usual care for a range of different ceiling ratios thereby showing decision uncertainty. Discounting is not necessary for the time horizon of 24 months.

### Data management

In compliance with the Dutch Personal Data Protection Act, all data are handled anonymized and confidentially. A study number will be given to all personal data of participants. Study reports, study documentation and publications will be using this study number and an independent researcher will be handling the key. Data will be collected and managed, during the study period, using GemsTracker electronic data capture tools hosted at Erasmus [23]. GemsTracker (GEneric Medical Survey Tracker) is a secure web-based application for distribution of questionnaires and forms during clinical research and quality registrations. Original paper case forms will be filed in the recruiting hospital’s investigator site file and entered in Gemstracker by the researcher. After the final data of the final included patient has been collected, all data will be stored for 15 years.

### Data monitoring

A data safety monitoring board is not required since the study is labeled as low risk. However, an independent monitoring board will at least monitor the study once a year. After all monitor visits a written report will be available.

A progress report will be submitted annually to the accredited Medical Research Ethics Committee by the investigator throughout the clinical trial. This report will consist of the number of subjects included, date of inclusion of the first subject, number of subjects that have completed the trial, (serious) adverse events ((S)AE’s), amendments and other problems.

As soon as the local researcher becomes aware of a(n) (S) AE this will be reported to the central researcher. A SAE will be reported via ToestingOnline within 7 days (death or life threatening situations) or 15 (remaining SAE’s) days by the central researchers.

### Dissemination

We plan to submit the manuscript to general peer-review journals and to present the study results at (inter) national conferences. We intent to implement the study results in the Dutch guideline for ACL injuries.

## Discussion

This study is a cluster randomized trial and therefore can be susceptible to selection bias [24]. In order to minimize this risk independent treating physicians are recruiting participants for this study. Because every physician from both intervention and control hospitals have been exposed to the same amount of trial training, the potential for selection bias is reduced.

This study is an open-label trial. The intervention to be investigated is visually different for the patient and treating physician. Randomization status will therefore not be blinded.

## Data Availability

The datasets used and/or analyzed during the current study are available from the corresponding author on reasonable request.

## Abbreviations

ACL: Anterior cruciate ligament
ACLR: Anterior cruciate ligament reconstruction
CONSORT: Consolidated Standards for Reporting Trials
SPIRIT: Standard Protocol Items Recommendations for Interventional Trials
MRI: Magnetic resonance imaging
PROM: Patient-rated outcome measures
IKDC: International Knee Documentation Committee
KOOS: Knee injury and Osteoarthritis Outcome Score
NRS: Number rating scale
EQ-5D-5L: EuroQol 5D
SDM-Q-9: 9-ltem Shared Decision Making Questionnaire
iMCQ: Medical Consumption Questionnaire
iPCQ: Production Consumption Questionnaire
QoL: Quality of life
CEA: Cost-effective analyses
CUA: Cost-utility analysis
ICER: Incremental cost-effectiveness ratio
QALY: Quality adjusted life years
AE: Adverse event
SAE: Serious adverse event
OR: Operating room
